# Therapeutic Potential of Arginine-Loaded Red Blood Cell Nanovesicles Targeting Obese Asthma

**DOI:** 10.1155/mi/8248722

**Published:** 2025-03-18

**Authors:** Quoc Quang Luu, Taejune Kim, Thi Bich Tra Cao, Injung Choi, Seung Yun Yang, Beum-Soo An, Dae Youn Hwang, Youngwoo Choi, Hae-Sim Park

**Affiliations:** ^1^Department of Oral and Maxillofacial Surgery, Loma Linda University School of Dentistry, Loma Linda, California, USA; ^2^Department of Biomaterials Science (BK21 FOUR Program), College of Natural Resources and Life Science, Pusan National University, Miryang, Republic of Korea; ^3^Department of Allergy and Clinical Immunology, Ajou University School of Medicine, Suwon, Republic of Korea

**Keywords:** arginine, asthma, microbiome, nanovesicles, obesity

## Abstract

**Purpose:** The role of the gut microbiomes has been emphasized in the pathogenesis of obese asthma (OA). However, the molecular mechanism of airway dysfunction underlying OA has not yet been fully elucidated. The effects of microbiomes on arginine metabolism in relation to lung functions and a novel method for delivering arginine to lung tissue based on arginine-loaded red blood cell (RBC)-derived nanovesicles (NVs) (NV^Arg^) will be investigated.

**Materials and Methods:** Inflammatory status, amino acid profiles, and microbial diversity were evaluated in 20 adult patients with OA compared to 30 adult patients with non-OA (NOA) and 10 healthy control (HC) groups. Changes in gut or lung microbial composition that altered arginine metabolism in relation to airway inflammation were investigated in an OA mouse model in vivo. Additionally, this study evaluated the delivery of arginine to lung tissue utilizing NV^Arg^ in vivo and in vitro.

**Results:** Significantly increased *Bacteroides* abundance but decreased serum arginine concentration with lower forced exhaled volume at 1 s (FEV_1_) (%) was noted in the OA group compared to the NOA and HC groups. In mouse experiments, when OA mice were given living bacteria from normal control (NC) mice, lung arginine concentration and airway resistance were restored. However, the administration of arginine or its metabolite (citrulline) did not increase the arginine levels in the lung tissues. We therefore created NV^Arg^, which successfully delivered arginine into the cytoplasm of the airway epithelial cell line *in vitro*. Oral administration of NV^Arg^ for OA mice significantly induced the AMP-activated protein kinase (AMPK) and endothelial nitric oxide synthase (eNOS) pathways in airway epithelial cells, which reduced airway resistance and inflammation.

**Conclusion:** These findings suggest that microbiomes contribute to airway dysfunction by regulating arginine metabolism, whereas NV^Arg^ treatment may be a potential option for managing OA.

## 1. Introduction

Obesity is a comorbidity of asthma, contributing to the development of asthma, where symptom severity increases with increasing body mass index (BMI) [1]. Previously, obese asthma (OA) was characterized by chronic low-grade inflammation consisting of increased CD8^+^ T cells, decreased regulatory T cells, and higher levels of circulating proinflammatory cytokines [2, 3]. Moreover, elevated reactive oxygen species have been known to be associated with the severity of OA [4]. However, it is not clear whether this airway inflammation directly induces airflow limitation, although obesity leads to a number of physiologic perturbations causing asthma symptoms [5].

Recently, emerging evidence has emphasized the function of the gut microbiome in the pathogenesis of asthma by regulating immune responses [6], epithelial integrity [7, 8], and lipid metabolism [9, 10]. To date, changes in the proportions of *Firmicutes* and *Bacteroidetes* have mainly been focused on the pathogenesis of obesity-related diseases [11, 12]. Furthermore, commensal bacteria have been shown to influence arginine metabolism involved in nitric oxide (NO) production [13], resulting in airway smooth muscle relaxation [14]. A central role of airway smooth muscles in the pathogenesis of airway dysfunction has been demonstrated [15]. Nevertheless, the underlying mechanisms by which commensal bacteria ameliorate airway dysfunction by modulating arginine metabolism have not been fully determined.

The metabolomic approach to identifying small biomolecules has provided insights into the pathophysiologic mechanisms of asthma [16–18]. A previous metabolomic study demonstrated that asthmatics with lower arginine levels have lower forced exhaled volume at 1 s (FEV_1_) (%) values [19]. In addition, NO synthase uncoupling due to an imbalance in arginine concentration has been demonstrated in OA. Therefore, arginine supplementation has beneficial effects on airway inflammation and dysfunction in asthmatic subjects, especially those who have lower or normal NO levels and type 2-low asthma [20–22]. Nevertheless, due to its significant first-pass metabolism in the liver and gut, its application as a therapeutic method is very restricted [23, 24]. In this aspect, red blood cell (RBC) nanovesicles (NVs) could be applicable for delivering drugs to specific cells or organs because these membrane vesicles have been recently shown to be safe and effective [25, 26].

Here, we hypothesized that the gut microbiome plays an important role in ameliorating airway dysfunction by regulating arginine production. The present study aimed to evaluate (1) the association between gut microbial proportion and amino acid profile in humans; (2) the role of the gut microbiome in the amelioration of airway dysfunction by regulating arginine metabolism in mice; and (3) the efficacy of arginine-loaded RBC-derived NVs (NV^Arg^) as an arginine carrier in targeted lung tissues in vivo and in vitro.

## 2. Materials and Methods

### 2.1. Patient Cohort and Characteristics

This study was approved by the Institutional Review Board of Ajou University Hospital (AJIRB-GEN-SMP-13-108). All patients provided written informed consent at the time of recruitment, and all experimental procedures involved in the study followed the ethical considerations of the Declaration of Helsinki. A BMI of more than 25 kg/m^2^ was considered obese, in accordance with the guidelines of the Korean Society for the Study of Obesity [27, 28]. The healthy control (HC) had no history of allergies or any other chronic diseases affecting asthma outcomes. Moreover, asthma was confirmed by an allergy specialist on the basis of clinical history, including recurrent cough, shortness of breath, recurrent wheeze, chest tightness, and evidence of airway obstruction, which was reversible after short-acting bronchodilator inhalation based on FEV_1_ (%) [29]. Spirometry was used to assess the degree of airway obstruction based on the Morris [30] method. The levels of total immunoglobulin E (IgE) were measured by the ImmunoCAP system (Thermo Fisher Scientific, Waltham, CA, USA). A hematology analyzer was used to determine blood total eosinophil count (TEC). Concentrations of serum arginine and citrulline were determined using an ELISA kit (MyBioSource, San Diego, CA, USA) according to the manufacturer's instructions.

### 2.2. Metagenomic 16S rRNA PCR Amplification and Sequencing

Bacterial DNA from human serum or mouse fecal/lung samples was extracted by using a PowerMax Soil DNA Isolation Kit (MO BIO Laboratories Inc., San Diego, CA, USA). Extracted DNA was purified with a silica-based spin filter FastDNA Kit (MP Biomedicals, Illkirch, France) and quantified using the Quant-iT PicoGreen dsDNA kit (Invitrogen, Waltham, MA, USA). Prepared DNA was used for PCR amplification of the V3–V4 hypervariable regions of the 16S ribosomal RNA genes using a primer set of 16S_V3_F (5ʹ-TCGTCGGCAGCGTCAGATGTGTATAAGAGACAGCCTACGGGNGGCWGCAG-3ʹ) and 16S_V4_R (5ʹ-GTCTCGTGGGCTCGGAGATGTGTATAAGAGACAGGACTACHVGGGTATCTAATCC-3ʹ) [31]. The PCR products were used to construct 16S rRNA gene libraries according to the MiSeq System guidelines (Illumina Inc., San Diego, CA, USA). The 16S rRNA gene libraries for individual samples were quantified using QIAxpert (QIAGEN, Hilden, Germany), pooled at an equimolar ratio, and used for pyrosequencing with the MiSeq System (Illumina Inc.) according to the manufacturer's recommendations.

### 2.3. Bacterial Diversity and Composition Analysis

Paired-end reads that matched the adapter sequences were trimmed by cutadapt (ver. 1.1.6) [32]. The resulting FASTQ files containing paired-end reads were merged using CASPER, and then the quality was filtered by the Phred (Q) score. Then, any reads under 350 bp or over 550 bp were discarded. Afterward, a reference-based chimera detection step was performed using VSEARCH against the SILVA gold database. The sequence reads were clustered into operational taxonomic units using the de novo clustering algorithm, and the threshold was 97% sequence similarity. Finally, operational taxonomic units were classified using UCLUST (*parallel_assign_taxonomy_uclust.py* script on QIIME version 1.9.1) under default parameters with the SILVA 128 database. Alpha diversity was determined by using the rarefaction curve of the Chao1 value.

### 2.4. Animal Study

All experimental protocols were approved by the Institutional Animal Care and Use Committee of Ajou University (IACUC 2021-0007) and performed in accordance with the Guide for the Care and Use of Laboratory Animals published by the Animal and Plant Quarantine Agency, Ministry of Agriculture, Food and Rural Affairs, Korea. In this study, female 6-week-old BALB/c mice (Orient BIO, Seongnam, Korea) were maintained under specific pathogen-free conditions. To establish the OA mouse model, mice were fed a high-fat diet (Research Diets, New Brunswick, NJ, USA) and provided 60% of energy in the form of fat for 6 months as previously described [24]. The establishment of OA was determined by airway dysfunction to inhaled methacholine (Sigma‒Aldrich) at a concentration of 50 mg/mL as measured using the FlexiVent System (SCIREQ, Montreal, Canada). For fecal transplantation, the fecal samples of normal control (NC) mice were harvested and suspended in 10% sterile glycerol to a final concentration of 50 mg/mL, and then mice received 1 mL of fecal transplant via rectal enema using a dissolution needle (Cadence Science, Cranston, RI, USA).

For comparison of efficacy between arginine and NV^Arg^ treatment, mice with OA were orally administered 10 mg/kg arginine or NV^Arg^ (0.1; 1; or 10 mg/kg) daily for 2 weeks. To observe the transfer of NV^Arg^ into each organ after oral administration, the pictures of NV^Arg^ stained with Cy7 mono NHS ester (GE Healthcare, Little Chalfont, UK) were obtained using an IVIS spectrum CT (SelectScience, Wilmslow, UK). In addition, the localization of NV^Arg^ in the lungs was confirmed by staining with DiI (Invitrogen). Fluorescence images were acquired using confocal laser scanning microscopy (Cal Zeiss Microscopy GmbH, Jena, Germany).

### 2.5. Measurement of Arginine and NO

Before harvesting the lung tissues, perfusion was performed through the mouse heart by inserting a needle into the left ventricle and injecting 5 mL of cold and sterile phosphate-buffered saline (PBS). When the lungs were extracted from mice, the weight was immediately measured. Then, the lungs were chopped with a blade and homogenized using FastPrep-24 (MP Biomedicals), followed by centrifugation at 1000× rpm for 10 min. The mixture was prepared by mixing equal volumes of supernatants and trichloroacetic acid, followed by centrifugation at 12,000× *g* for 15 min. The levels of arginine and NO were measured in the lung tissues by using kits (MyBioSource) according to the manufacturer's instructions.

### 2.6. NV^Arg^ Preparation and Characterization

Blood samples (10 mL) from NC mice were collected in BD Vacutainer tubes containing acid citrate dextrose solution (BD Biosciences, Franklin Lakes, NJ, USA). To isolate RBC, the samples were layered on a Lymphoprep (Axis-Shield, Oslo, Norway), followed by centrifugation at 879× *g* for 25 min without stopping. Then, RBCs were washed with 1 × PBS three times and recollected by centrifugation at 800× *g* for 5 min at 4°C. To prepare NVs, the supernatants were extruded through 400-nm polycarbonate porous membranes with a mini-extruder (Avanti Polar Lipids, Birmingham, AL, USA). To encapsulate arginine into NVs, 10 mg/mL arginine (Sigma‒Aldrich) was mixed with NVs. Finally, the mixture was extruded through 400-nm polycarbonate porous membranes with a mini-extruder three times and centrifuged with a type 90 Ti Fixed-Angle Titanium Rotor (Beckman Coulter, Fullerton, CA, USA) at 120,000× *g* for 2 h at 4°C. The purified NV^Arg^ was stored in PBS at −80°C. The shape of NV^Arg^ was determined through a transmission electron microscope (JEM1011; JEOL, Akishima, Japan). The size of NV^Arg^ was measured using a Zetasizer Nano S (Malvern Instruments, Malvern, UK). Protein bands were visualized by sodium dodecyl sulfate‒polyacrylamide gel electrophoresis. The abundance of arginine in NV^Arg^ was determined using the arginine ELISA Kit (MyBioSource).

### 2.7. In Vitro Experiment

To investigate the effect of NV^Arg^ on human airway epithelial cells, A549 cells (American Type Culture Collection, Manassas, VA, USA) were purchased and cultured in RPMI medium (Sigma‒Aldrich) with 10% fetal bovine serum, 100 IU/mL penicillin, and 50 μg/mL streptomycin at 37°C with 5% CO_2_ in humidified air. For NV^Arg^ treatment, the cells were maintained in serum-free RPMI without arginine (Sigma‒Aldrich) and stimulated with 10 μg/mL NV^Arg^ (total protein) for 48 h. To observe the uptake of NV^Arg^ into the cells, A549 cells were treated with or without 1 μM DiO (Invitrogen)-labeled NV^Arg^ for 6 h. Finally, the cells were coated with 4′,6-diamidino-2-phenylindole (DAPI; 1:1000) for 5 min. Cell viability was evaluated using the Cell Counting Kit-8 (Sigma‒Aldrich). Moreover, NO and interleukin (IL)-8 concentrations in the cell culture supernatants were measured using NO Assay Kits (MyBioSource) and IL-8 ELISA kits (R&D Systems) according to the manufacturer's instructions.

### 2.8. Western Blotting

Anti-arginase (Invitrogen), anti-ornithine transcarbamylase (1:1000, Abcam, Cambridge, MA, USA), anti-argininosuccinate synthase (1:1000, Abcam), anti-argininosuccinate lyase (1:1000, Abcam), and anti-actin antibodies (1:1000, Santa Cruz, Dallas, TX, USA) were used. Anti-AMP-activated protein kinase ([AMPK], 1:1000, Cell Signaling; Danvers, MA, USA), anti-endothelial NO synthase (eNOS, 1:1000, Cell Signaling), anti-pAMPK (1:1000, Cell Signaling), and anti-peNOS antibodies (1:1000, Invitrogen) were used.

### 2.9. Statistical Analysis

Normality tests were performed with Shapiro‒Wilk normality tests. Statistical analyses were performed using GraphPad Prism software version 8.0.2 (GraphPad Inc., San Diego, CA, USA) and SPSS software version 26.0 (IBM Corp., Armonk, NY, USA). Differences between two continuous variables were assessed by using the Student's *t* test for normally distributed variables or by the Mann‒Whitney *U* test for nonnormally distributed variables. Multiple groups were compared by one-way ANOVA with Bonferroni's post hoc test for normally distributed variables or by the Kruskal‒Wallis test with Dunn's post hoc test for nonnormally distributed variables. Associations between two independent variables were represented as the Pearson or Spearman correlation coefficient *r* and evaluated by the two-tailed test. Multivariable logistic regression models examined associations between OA and related factors. When comparing OA-related factors between OA and other groups (HCs and non-OA [NOA]), the generalized linear model was applied to adjust for age as a covariate. The differences were considered significant if the *p* value was lower than 0.05.

## 3. Results

### 3.1. Decreased Serum Arginine Concentration in Patients With OA

The demographic data of the study subjects are depicted in Table 1. Among patients with OA, BMI was significantly higher than in HC and those with NOA (*p* < 0.001 for all). Nevertheless, the mean of baseline FEV_1_% was notably lower in patients with OA compared to both the HC group and patients with NOA (84.1 ± 17.6 *vs*. 109.5 ± 11.5, *p* < 0.001; 84.1 ± 17.6 *vs*. 95.2 ± 13.3, *p*=0.033; respectively). Moreover, significantly decreased levels of serum arginine were noted in patients with OA compared to HCs (*p*=0.001). Serum arginine levels were lower in OA patients than in NOA patients; however, no statistically significant difference was observed (*p* =0.121). Other characteristics, including the prevalence of current smoking (*p* > 0.05 for all) and TEC (*p* > 0.05 for all), were not significantly different between the three groups.

The predictability of each clinical parameter for distinguishing the OA group from another group (HCs and patients with NOA) was examined using univariate and multivariate logistic regression analyses (Table 2). In the univariate analysis, significant parameters associated with OA included age, serum arginine levels, and FEV_1_ (*p* < 0.050). However, the multivariate analysis revealed that only serum arginine levels and FEV_1_ remained significant parameters for predicting OA (odds ratio [OR] = 0.948, 95% confidence interval [95%CI] = 0.901–0.997, *p*=0.040, for arginine, and OR = 0.936, 95%CI = 0.889–0.985, *p*=0.011, for FEV_1_). These findings suggest that metabolic changes in arginine production may play a role in the development of OA, contributing to the decline in lung function.

### 3.2. Association Between Microbiome and Metabolic Status in Humans

**Figure 1:**
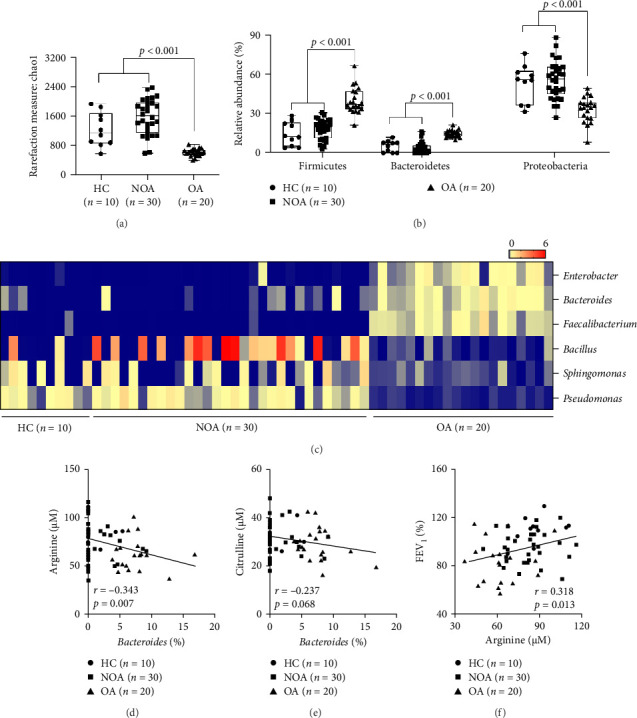
Microbial diversity and composition in blood samples of the study subjects. (A) Chao1 diversity index. (B) Microbial composition at the phylum level. (C) Heatmap plot of the microbial communities at the genus level. (D and E) Correlations between the relative abundance of *Bacteroides* and arginine/citrulline concentration. (F) A positive correlation between arginine concentration and FEV_1_%. The data are presented as the Pearson's correlation coefficient *r* (*p* value). Horizontal lines indicate median values, and whiskers indicate minimum to maximum values. *p* values were adjusted for age as a covariate using a generalized linear model. HC, healthy control; NOA, non-obese asthma; OA, obese asthma.

**Figure 2:**
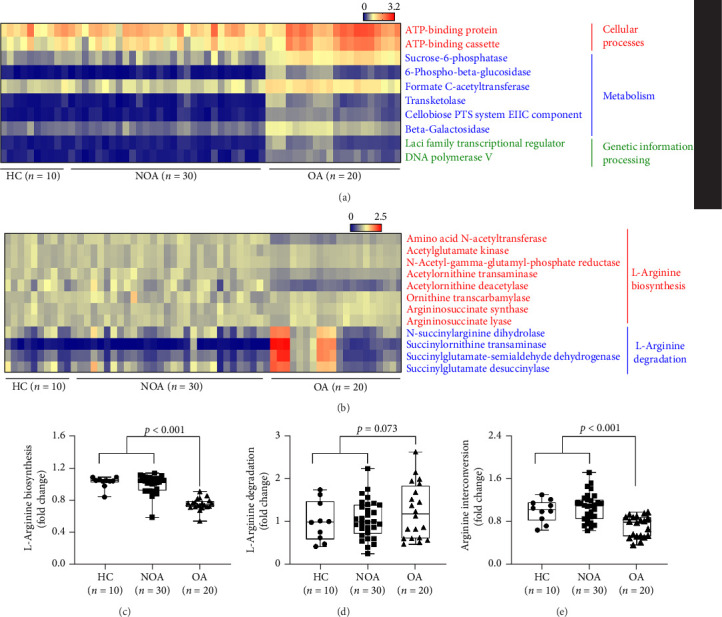
Functional gene profiles of the microbiome based on metagenomic analysis. (A) Heatmap plot of multiple genes associated with cellular processes, metabolism, and genetic information processing. (B) Evaluation of the relative abundance of microbial genes related to L-arginine biosynthesis and L-arginine degradation involved in arginine metabolism. (C–E) Genes contributing to L-arginine biosynthesis and L-arginine degradation as well as arginine, ornithine, and proline interconversion. Horizontal lines indicate median values, and whiskers indicate minimum to maximum values. *p* values were adjusted for age as a covariate using a generalized linear model. AST, arginine succinyltransferase; ATP, adenosine triphosphate; HC, healthy control; NOA, non-obese asthma; OA, obese asthma.

The present study conducted metagenomic analysis to confirm changes in microbial diversity and composition in the study subjects. In the OA group, markedly reduced microbial diversity was observed compared to another group (HC and NOA) (*p* < 0.001; Figure 1A). Moreover, higher proportions of *Bacteroidetes* and *Firmicutes*, but a lower proportion of *Proteobacteria*, were noted in patients with OA (*p* < 0.001 for all; Figure 1B). At the genus level, the relative abundances of *Enterobacter*, *Bacteroides*, and *Faecalibacterium* were significantly higher, whereas those of *Bacillus*, *Sphingomonas*, and *Pseudomonas* were significantly lower in patients with OA (Figure 1C). Among them, the proportion of *Bacteroides* was significantly correlated with serum arginine but not citrulline concentration (*r* = −0.343, *p*=0.007 for arginine, and *r* = −0.237, *p*=0.068 for citrulline; Figure 1D,E). In addition, arginine concentration had a positive correlation with baseline FEV_1_ (%) (*r* = 0.318, *p*=0.013; Figure 1F). In addition, several proteins involved in cellular processes, metabolism, and genetic information processing were identified (Figure 2A,B). Especially, enzyme expressions involved in L-arginine biosynthesis as well as in arginine interconversion, but not in L-arginine degradation, were significantly different between two groups (*p* < 0.001, *p* < 0.001, and *p*=0.073, respectively; Figure 2).

### 3.3. Comparison Between Gut and Lung Microbiome in Mice

To identify causative factors contributing to changes in arginine production, this study analyzed the alteration of microbial diversity and composition in mice with or without OA. In the guts, significantly lower species richness was noted in mice with OA compared with NC mice (*p*=0.001, Figure S1A). Moreover, the microbial composition was markedly different between two groups (Figure S1B). Especially, the relative abundance of *Lactobacillus* decreased, but that of *Bacteroides* increased in mice with OA at the genus level (Figure S1C). Furthermore, arginine concentration was positively correlated with the proportion of *Lactobacillus* but negatively correlated with the proportion of *Bacteroides* (*r* = 0.659, *p*=0.038, for *Lactobacillus*; *r* = −0.701,*p*=0.024, for *Bacteroides*; Figure S1D). However, significant changes in the lung microbiome were not observed in contrast to the gut microbiome (Figure S2A–D), suggesting that the gut microbiome may be involved in arginine production rather than the lung microbiome.

### 3.4. Effects of the Gut Microbiome on Arginine Production and Lung Function in Mice

**Figure 3:**
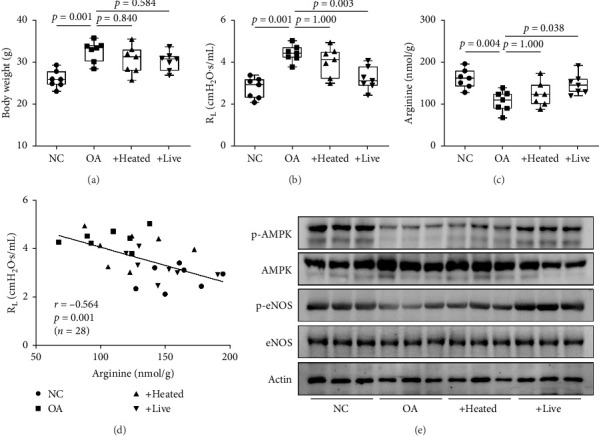
Role of the gut microbiome in arginine metabolism in obese asthma (OA). Live or heated bacteria were isolated from normal control (NC) mice and transplanted into mice with OA. (A) Changes in the body weight of mice. (B) Changes in airway resistance (*R*_L_). (C) Arginine concentration in the lung tissues. (D) Correlations between airway resistance and arginine concentration in the lungs. The data are presented as the Pearson's correlation coefficient *r* (*p* value). (E) Phosphorylation of AMPK and eNOS. Horizontal lines indicate median values, and whiskers indicate minimum to maximum values. *p* values were determined by the one-way ANOVA with Bonferroni's post hoc test. AMPK, AMP-activated protein kinase; eNOS, endothelial nitric oxide synthase.

To investigate the function of the gut microbiome in OA, live or heated bacteria obtained from NC mice were administered to mice with OA. When fecal transplantation was performed, body weight was not significantly decreased in mice with OA (*p* > 0.050 for all, Figure 3A); however, airway resistance was markedly ameliorated by administration of live but not heated bacteria (*p*=0.003 for living bacteria and *p*=1.000 for heated bacteria; Figure 3B). Moreover, the arginine concentration was significantly elevated in the lungs by living bacteria (*p*=0.038, Figure 3C). Furthermore, the degree of airway resistance showed a negative correlation with arginine levels (*r* = −0.564, *p*=0.001, Figure 3D). In the current study, we observed increased phosphorylation of AMPK and eNOS in the lung tissues by administration of live bacteria (Figure 3E). Moreover, arginase was highly expressed in the lung tissues in OA mice, which was not reduced by live or heated bacteria. Moreover, the expression of other proteins, including ornithine transcarbamylase, argininosuccinate synthase, or argininosuccinate lyase, was not significantly changed by the administration of bacteria (Figure S3), suggesting that the gut microbiome may influence arginine production but not the activation of host enzymes.

### 3.5. NV^Arg^ Preparation, Characterization, and Treatment

**Figure 4:**
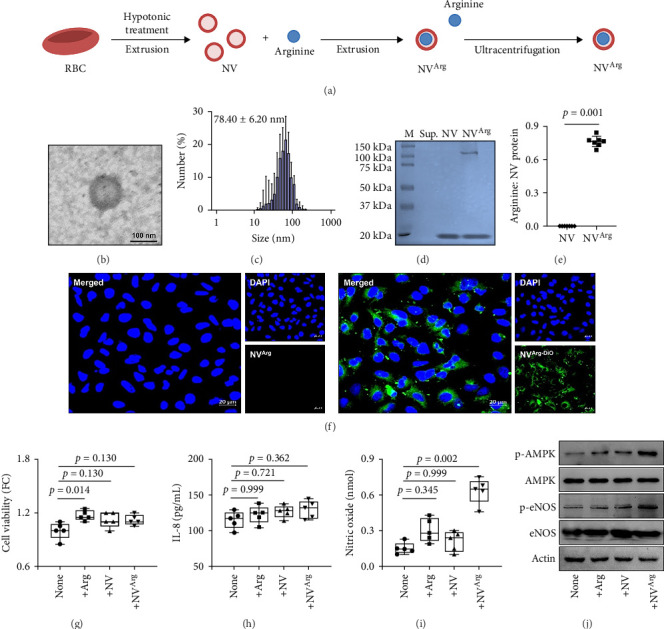
Effect of NV^Arg^ on airway epithelial cells to produce NO via the AMPK–eNOS pathway. (A) Schematic protocol of NV^Arg^ construction. (B) Transmission electron microscopic images of NV^Arg^. Scale bar, 100 nm. (C) Size of NV^Arg^ measured using dynamic light scattering. (D) Protein band patterns of NV and NV^Arg^. (E) Ratio of arginine to the total protein concentration of NV^Arg^. (F) Confocal images of airway epithelial cells treated with or without NV^Arg-DiO^. (G) Changes in cell viability after NV^Arg^ treatment. (H and I) The levels of IL-8 and nitric oxide produced by airway epithelial cells. (J) The expression of phosphorylated AMPK and eNOS in airway epithelial cells. Horizontal lines indicate median values, and whiskers indicate minimum to maximum values. *p* values were determined by the Mann–Whitney *U* test or by the one-way ANOVA with Bonferroni's post hoc test or by the Kruskal–Wallis with Dunn's post hoc test. Arg, arginine; DAPI, 4′,6-diamidino-2-phenylindole; eNOS, endothelial nitric oxide synthase; IL, interleukin; MPK, AMP-activated protein kinase; NV, RBC-derived nanovesicles; NV^Arg^, arginine-loaded RBC-derived nanovesicles; NV^Arg-DiO^, DiO-labeled NVArg; RBC, red blood cell; Sup, supernatant.

The schematic protocol of NV^Arg^ construction is shown in Figure 4A. When NV^Arg^ was observed by transmission electron microscopy, these spherical membrane vesicles were delimited by a lipid bilayer with a size range from 70 to 90 nm (Figure 4B,C). Moreover, NV and NV^Arg^ could be distinguished by sodium dodecyl sulfate‒polyacrylamide gel electrophoresis. NV^Arg^ showed only one different protein band between 100 and 150 kDa compared to NV without arginine (Figure 4D). To determine the total protein concentration of NV^Arg^ for *in vitro* and *in vivo* experiments, the ratio of arginine to total protein was evaluated. As a result, ~0.8 μg/mL arginine was contained per 1 μg/mL total protein of NV^Arg^ (Figure 4E). To investigate whether NV^Arg^ could affect airway epithelial cells by uptake in the cells, NV^Arg^ was stained with DiO, which is a green fluorescent lipophilic carbocyanine dye widely used as a lipophilic tracer. When airway epithelial cells were treated with DiO-labeled NV^Arg^, fluorescence was detected in the cytoplasm rather than the nucleus of the cells (Figure 4F). In addition, NV or NV^Arg^ treatment did not show any cytotoxicity because no significant differences were noted in cell viability and IL-8 production (*p* > 0.050 for all; Figure 4G,H). As NO production is known to be associated with muscle relaxation, the present study further evaluated the NO concentration released from airway epithelial cells with or without NV^Arg^ treatment. As a result, NV^Arg^ significantly enhanced NO production from the cells (*p*=0.002); however, arginine and NV could not stimulate airway epithelial cells (*p*=0.345 for arginine and *p*=0.999 for NV; Figure 4I). Moreover, NV^Arg^ induced phosphorylation of AMPK and eNOS in the cells in a dose-dependent manner (Figure 4J).

### 3.6. The Effect of NV^Arg^ on Airway Resistance in OA Mice

**Figure 5:**
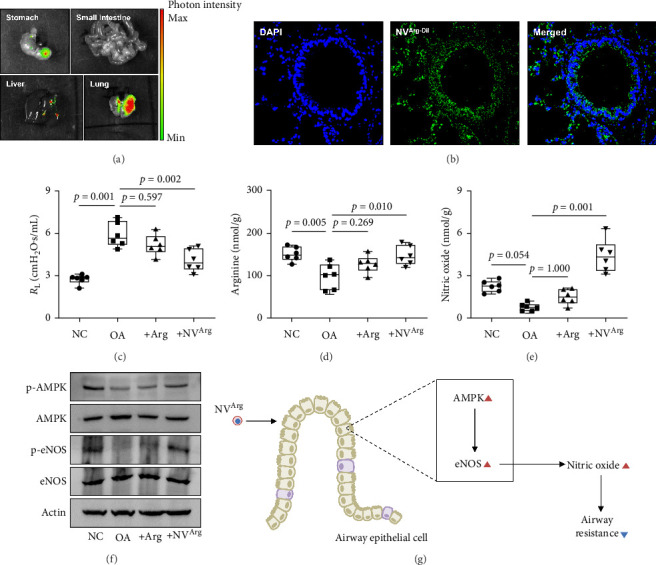
Efficacy of NV^Arg^ in mice with obese asthmatic mice compared to normal control mice. (A) Fluorescence assay for the detection of NV^Arg-Cy7^ in dissected organs after oral administration. (B) The presence of NV^Arg-DiI^ in the lung tissues of mice. (C) Changes in airway resistance (*R*_L_). (D) The levels of arginine in the lungs. (E) Nitric oxide production in the lungs. (F) Phosphorylation of AMPK and eNOS in the lungs. (G) The proposed mechanisms by which NV^Arg^ reduces airway resistance in OA via the AMPK–eNOS pathway with nitric oxide production. Horizontal lines indicate median values, and whiskers indicate minimum to maximum values. *p* values were determined by one-way ANOVA with Bonferroni's post hoc test or Kruskal–Wallis with Dunn's post hoc test. AMPK, AMP-activated protein kinase; Arg, arginine; DAPI, 4′,6-diamidino-2-phenylindole; eNOS, endothelial nitric oxide synthase; NV^Arg^, arginine-loaded RBC-derived nanovesicles; NV^Arg-DiI^, DiI-labeled NV^Arg^; NVs, RBC-derived nanovesicles; RBC, red blood cell.

When NC mice were orally treated with NV^Arg^, these membrane vesicles were absorbed from the stomach and detected in the lungs rather than other organs including the small intestine (Figure 5A,B). Furthermore, the present study compared the efficacy of arginine and NV^Arg^ treatment. The results showed that NV^Arg^, but not arginine, significantly reduced airway resistance in mice with OA (*p*=0.002; Figure 5C). Moreover, NV^Arg^ treatment markedly enhanced arginine and NO production in the lungs (*p*=0.010 for arginine and *p*=0.001for NO; Figure 5D,E). By western blot analysis, the function of NV^Arg^ in the AMPK–eNOS pathway was confirmed (Figure 5F). Taken together, we suggest a schematic picture of how NV^Arg^ attenuates airway resistance in OA, as depicted in Figure 5G.

## 4. Discussion

This is the first study to demonstrate the significance of the gut microbiome in association with amino acid metabolism in patients/mice with OA. Especially, mice with OA had a higher proportion of *Bacteroides* but a lower proportion of *Lactobacillus* in the gut. In addition, decreased arginine concentration and airway dysfunction were noted in mice with OA after receiving live bacteria obtained from NC mice. Furthermore, we developed NV^Arg^ and found that these novel molecules could attenuate airway inflammation and dysfunction by NO production (AMPK–eNOS pathway) in vitro and in vivo. Taken together, these results provide new insight into the mechanism of OA by altering the gut microbial composition and amino acid metabolism, in which the application of NVs for drug delivery systems could serve as new insight into controlling the symptoms of OA.

The altered gut microbial composition has been associated with obesity-related diseases because the largest source of microbial exposure in humans comes from the intestinal tract, which contains a diverse population of microbes [33, 34]. The family *Bacteroidetes* is composed of gram-negative bacteria predominantly found in obese subjects; however, the role of this microorganism remains controversial [11, 12]. In particular, the family *Bacteroidetes* promotes carbohydrate fermentation, resulting in a pool of volatile fatty acids that are reabsorbed into the large intestine. Nonetheless, its virulence, capsule, and enterotoxin might elicit an immunological response in the host [11, 12]. In the present study, the proportion of *Bacteroidetes* in humans was higher in patients with OA than HC, which was consistent with the results of a study by Andreas et al. but differed from those of other studies demonstrating that the proportion of *Bacteroidetes* was lower in obese subjects than in lean subjects [35, 36]. These findings were supported by *in vivo* experiments in which a higher proportion of *Bacteroides* was found in the gut of obese asthmatic mice than in NC mice. In contrast to *Bacteroides*, *Lactobacillus*, which was decreased in obese asthmatic mice, has favorable benefits in the restoration of T_h_2/T_h_1 immunological imbalance as well as of CD4^+^CD25^+^Foxp3^+^ T_reg_ functions in the lungs, thereby ameliorating airway dysfunction and inflammation [37]. These findings imply that a higher proportion of *Bacteroides* and a lower proportion of *Lactobacillus* could lead to poor clinical outcomes of OA.

Changes in amino acid metabolism could be a possible mechanism related to the development of multiple diseases in obese subjects, including asthma [20–22]. In particular, arginine balance has been reported to be involved in lung function decline and the development of respiratory symptoms in asthmatic patients [38]. In the present study, we observed lower arginine concentrations in patients/mice with OA. Additionally, arginine is hydrolyzed to urea and ornithine depending on arginase activity in the urea cycle. Therefore, a lack of arginine leads to NO deficiency because NOS cannot sufficiently use arginine as a substrate [20–22]. Moreover, three NOS isozymes differ in structure and origin as follows: inducible NOS (iNOS) from activated macrophages, eNOS from endothelial cells, and neural NOS from neurons. Functionally, high NO synthesis by iNOS is largely important for preventing invading pathogens, whereas NO pulses by eNOS induce low NO waves that sustain smooth muscle relaxation. The decreased production of NO in mice with asthma could be attributable to the low expression of eNOS, which is consistent with the results of previous studies [39]. Therefore, decreased NO production, rather than immune cell activation and airway function, may be responsible for restricted smooth muscle relaxation in OA.

The current therapeutics for controlling OA phenotypes, such as nonsurgical and surgical weight loss approaches, significantly enhance symptomatic control and spirometric lung function; however, they have drawbacks related to invasive procedures, adverse reactions, and difficulty with maintenance over many years [40]. Moreover, inverse correlations between BMI and steroid responsiveness (the first-line anti-inflammatory medication for asthmatics) were noted in an in vitro study [41]. To address these issues for the management of OA, arginine supplementation was suggested as an efficient and cost-effective strategy for the prevention and treatment of metabolic syndrome, which includes obesity and asthma [42]. Arginine supplementation was previously performed to control asthmatic symptoms; however, the efficacy of arginine remains uncertain, as this molecule is mostly hydrolyzed to ornithine after uptake in the small intestine [20–22]. When our OA mice were orally administered arginine, both the levels of arginine in the lung and the degree of airway dysfunction were affected. Taken together, arginine administration alone could be insufficient to restore arginine levels in lung tissue. As drug delivery systems, membrane-bound organelles called extracellular vesicles (EVs) have represented an endogenous mechanism for intercellular communication due to their function of transferring biological information [20–22, 43]. Moreover, EVs derived from RBC have been suggested for therapeutic applications in drug delivery systems [44]. In light of such advantages, we developed NV^Arg^, which could efficiently transfer from the gut to the lungs by absorbance in the stomach instead of going down to the small intestine. As our expectation, treatment with NV^Arg^ has been shown to more effectively improve airway dysfunction in mice with OA than arginine-alone supplementation.

Nevertheless, the present study does have some limitations. Firstly, to gain more direct and valid evidence of microbiome changes in patients with OA, it would be beneficial to evaluate the microbiome composition in the gut or respiratory tracts. Secondly, since this is an observational study, conducting large-scale and multicenter studies would be necessary to confirm our results. Moreover, the precise mechanisms governing the absorption of NVs from the stomach and their subsequent detection in the lungs instead of other organs remain unclear. Further research is necessary to elucidate these mechanisms. Finally, other confounding variables (medication usage and diet of patients), which may alter microbial diversity regardless of illness condition, were not included in our study. These variables should be examined in future studies. Despite these limitations, changes in the gut microbiome and arginine metabolism may contribute to the pathogenesis of OA by reducing NO production via the AMPK–eNOS pathway. Therefore, modulation of arginine metabolism can be a new therapeutic target for patients with OA whose condition is not fully controlled by conventional treatment. In addition, NV^Arg^ could be considered a therapeutic agent for better control of OA by enhancing NO production via the AMPK–eNOS pathway in the lungs, restoring airway inflammation and dysfunction.

## Figures and Tables

**Table 1:** Demographic data of the study subjects.

Variables	HC (*n* = 10)	NOA (*n* = 30)	OA (*n* = 20)	*p* value (HC vs. NOA)	*p* value (HC vs. OA)	*p* value (NOA vs. OA)
Age (years)	25.0 (24.8–26.0)	45.0 (36.5–55.0)	53.0 (42.0–61.0)	<0.001	<0.001	0.502
Sex (female, %)	3/10 (30.0)	19/30 (63.3)	11/20 (55.0)	0.067	0.196	0.556
BMI (kg/m^2^)	21.6 ± 1.7	21.9 ± 2.0	28.0 ± 2.6	1.000	<0.001	<0.001
Smoking (*n*, %)	2/10 (20.0)	8/30 (26.7)	5/20 (25.0)	0.673	0.760	0.895
FEV_1_ (%)	109.5 ± 11.5	95.2 ± 13.3	84.1 ± 17.6	0.029	<0.001	0.033
Total IgE (kU/L)	41.0 (6.5–164.5)	197.5 (54.5–553.3)	141.0 (58.5–209.3)	0.033	0.207	1.000
TEC (cells/μL)	200.0 (100.0–225.0)	406.7 (105.3–868.3)	248.0 (54.4–501.5)	0.198	1.000	0.625
Arginine (μM)	88.0 ± 13.8	73.1 ± 18.6	62.8 ± 15.9	0.061	0.001	0.121
Citrulline (μM)	33.0 ± 5.4	30.2 ± 7.2	29.5 ± 6.9	0.802	0.556	1.000

*Note:* Values are given as *n* (%) for categorical variables, as the mean ± standard deviation for normally distributed variables, and as the median for nonnormally distributed variables. *p* values were obtained by Pearson's chi-squared test for categorical variables or one-way ANOVA with post hoc Bonferroni for normally distributed variables or Kruskal‒Wallis test with post hoc Dunn's test for nonnormally distributed variables.

Abbreviations: BMI, body mass index; FEV_1_, forced exhaled volume at 1 s; HC, healthy control; IgE, immunoglobulin E; NOA, non-OA; OA, obese asthma; TEC, total eosinophil count.

**Table 2:** Predictability for an obese group (obese asthma) from non-obese groups (healthy controls and non-obese asthma) by univariate and multivariate logistic regression analyses.

Variables	Univariate			Multivariate		
	Odds ratio	95%CI	*p* value	Odds ratio	95%CI	*p* value
Age (years)	1.058	1.014–1.104	0.009	—	—	—
Sex (male/female)	1.000	0.340-2.942	1.000	—	—	—
Smoking (current/others)	1.000	0.290–3.454	1.000	—	—	—
FEV_1_ (%)	0.941	0.904–0.980	0.003	0.936	0.889–0.985	0.011
Total IgE (kU/L)	0.999	0.997–1.001	0.448	—	—	—
TEC (cells/μL)	0.999	0.998–1.001	0.333	—	—	—
Arginine (μM)	0.955	0.922–0.989	0.009	0.948	0.901–0.997	0.040
Citrulline (μM)	0.968	0.893–1.050	0.436	—	—	—

Abbreviations: FEV_1_, forced exhaled volume at 1 s; HC, healthy control; IgE, immunoglobulin E; OA, obese asthma; TEC, total eosinophil count.

## Data Availability

The data that support the findings of this study are available upon reasonable request from the corresponding author.

## Supporting Information

Supporting Information Figure S1: comparison of microbiome in gut between normal control (NC) and mice with obese asthma (OA). Figure S2: comparison of microbiome in lungs between normal control (NC) and mice with obese asthma (OA). Figure S3: expression of multiple proteins involved in arginine metabolism.
