# Supplementary material for: Therapeutic Potential of Arginine-Loaded Red Blood Cell Nanovesicles Targeting Obese Asthma
Source: Mediators Inflamm. 2025 Mar 18;2025:8248722. doi: 10.1155/mi/8248722 (PMC11936518; doi:10.1155/mi/8248722)
Supplement: Supporting Information — Figure S1: comparison of microbiome in gut between normal control (NC) and mice with obese asthma (OA). Figure S2: comparison of microbiome in lungs between normal control (NC) and mice with obese asthma (OA). Figure S3: expression of multiple proteins involved in arginine metabolism. [file 8248722.f1.docx]

**Supplementary Figures**

**Therapeutic potential of arginine-loaded red blood cell nanovesicles targeting obese asthma**

**Running title: Arginine-loaded nanovesicles in obese asthma**

Quoc Quang Luu,^1*^ Taejune Kim,^2*^ Thi Bich Tra Cao,^3^ Injung Choi,^2^ Seung Yun Yang,^2^ Beum-Soo An,^2^ Dae-Youn Hwang,^2^ Youngwoo Choi,^2#^ Hae-Sim Park^3#^

^1^Department of Oral & Maxillofacial Surgery, Loma Linda University School of Dentistry, CA, USA

^2^Department of Biomaterials Science (BK21 FOUR program), College of Natural Resources and Life Science, Pusan National University, Miryang, Korea

^3^Department of Allergy and Clinical Immunology, Ajou University School of Medicine, Suwon, Korea

*These authors equally contributed to this work

#These authors are co-corresponding authors

**Corresponding authors:**

Youngwoo Choi, PhD

Department of Biomaterials Science, College of Natural Resources and Life Science, Pusan National University, 1268-50 Samnangjin-ro, Miryang, Korea

Tel: +82-55-350-5385;

Fax: +82-55-350-5389;

Email: ychoi@pusan.ac.kr

Hae-Sim Park, MD, PhD

Department of Allergy and Clinical Immunology, Ajou University School of Medicine, Ajou University Medical Center, 164 Worldcup-ro, Suwon, Korea

Tel: +82-31-219-4779;

Fax: +82-31-219-5154;

Email: hspark@ajou.ac.kr


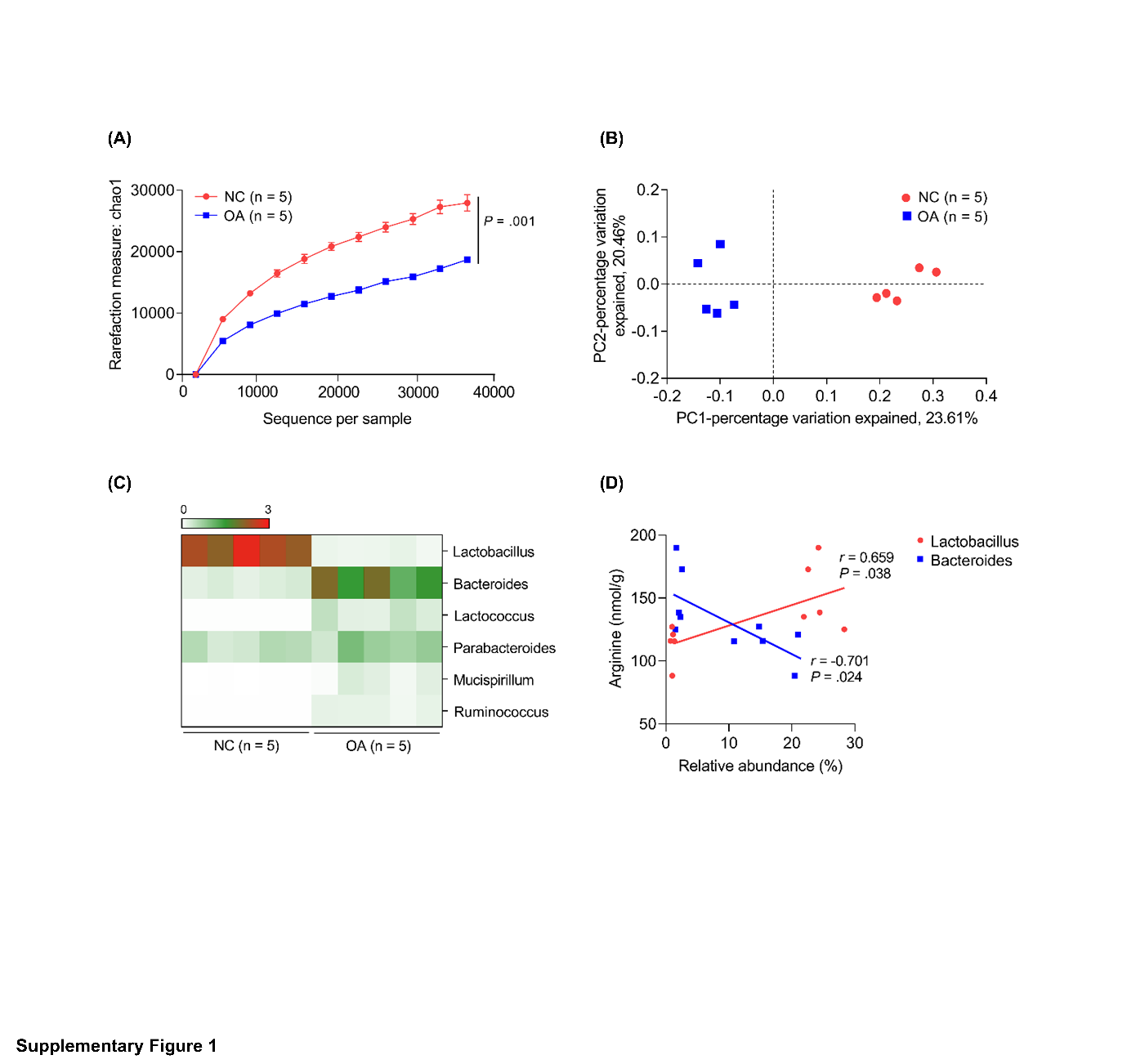
**Supplementary Figure 1:** Comparison of microbiome in gut between normal control (NC) and mice with obese asthma (OA). (A) Chao1 diversity index. (B) Analysis of the variance between microbial communities from the guts evaluated by the average relative abundance using principal component analysis. (C) Heatmap plot of the gut microbiota at the genus level. (D) Correlations between arginine concentration and relative abundance of *Lactobacillus*/*Bacteroides*. The data are represented as Pearson correlation coefficient r (*P* value). *P* values were determined by the Mann–Whitney *U* test. PC, principal component.


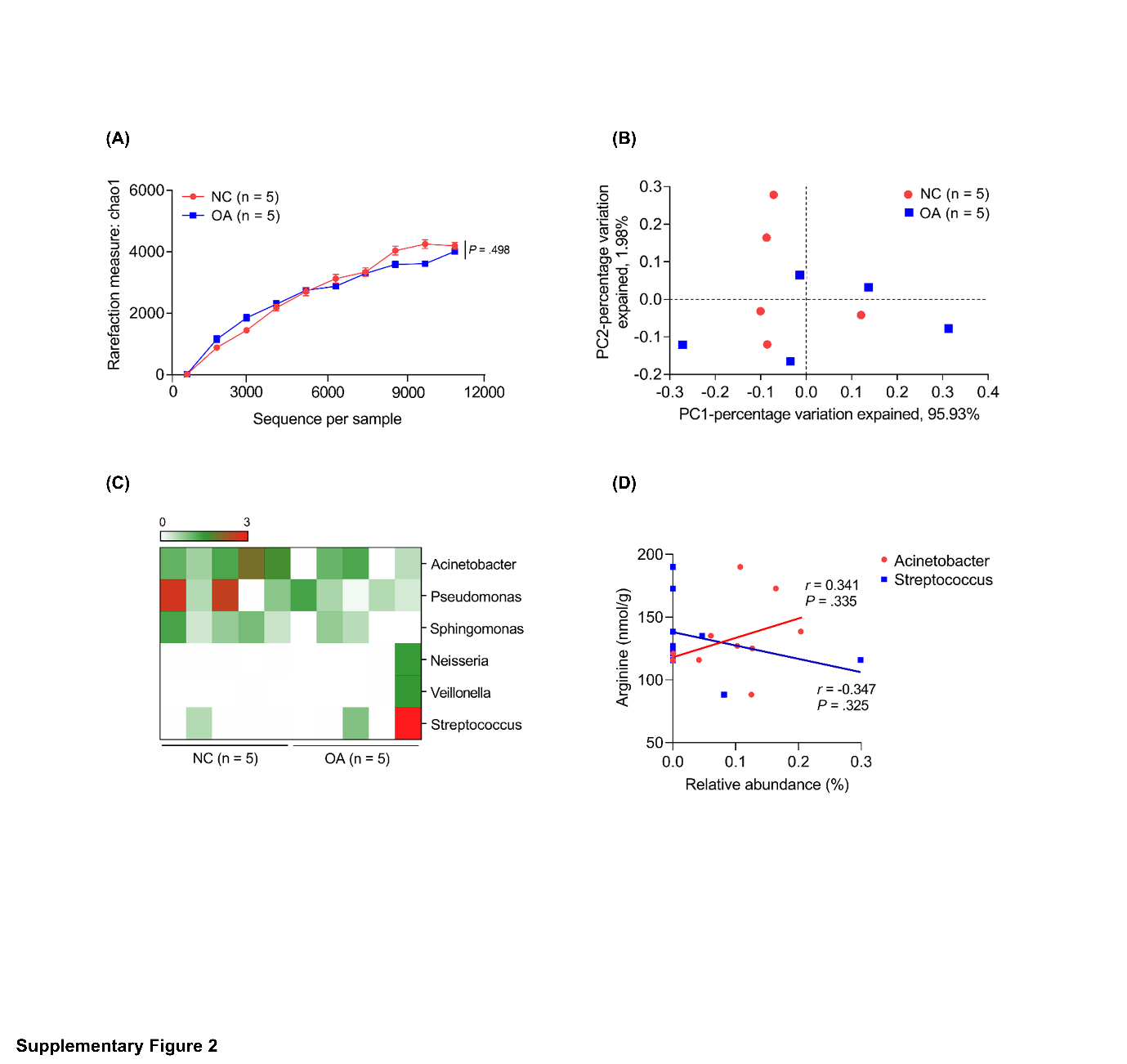
**Supplementary Figure 2:** Comparison of microbiome in lungs between normal control (NC) and mice with obese asthma (OA). (A) Chao1 diversity index. (B) Analysis of the variance between microbial communities from the lungs evaluated by the average relative abundance using principal component analysis. (C) Heatmap plot of the gut microbiota at the genus level. (D) Correlations between arginine concentration and relative abundance of Acinetobacter/Streptococcus. The data are represented as Pearson correlation coefficient r (*P* value). *P* values were determined by the Mann–Whitney *U* test. PC, principal component.


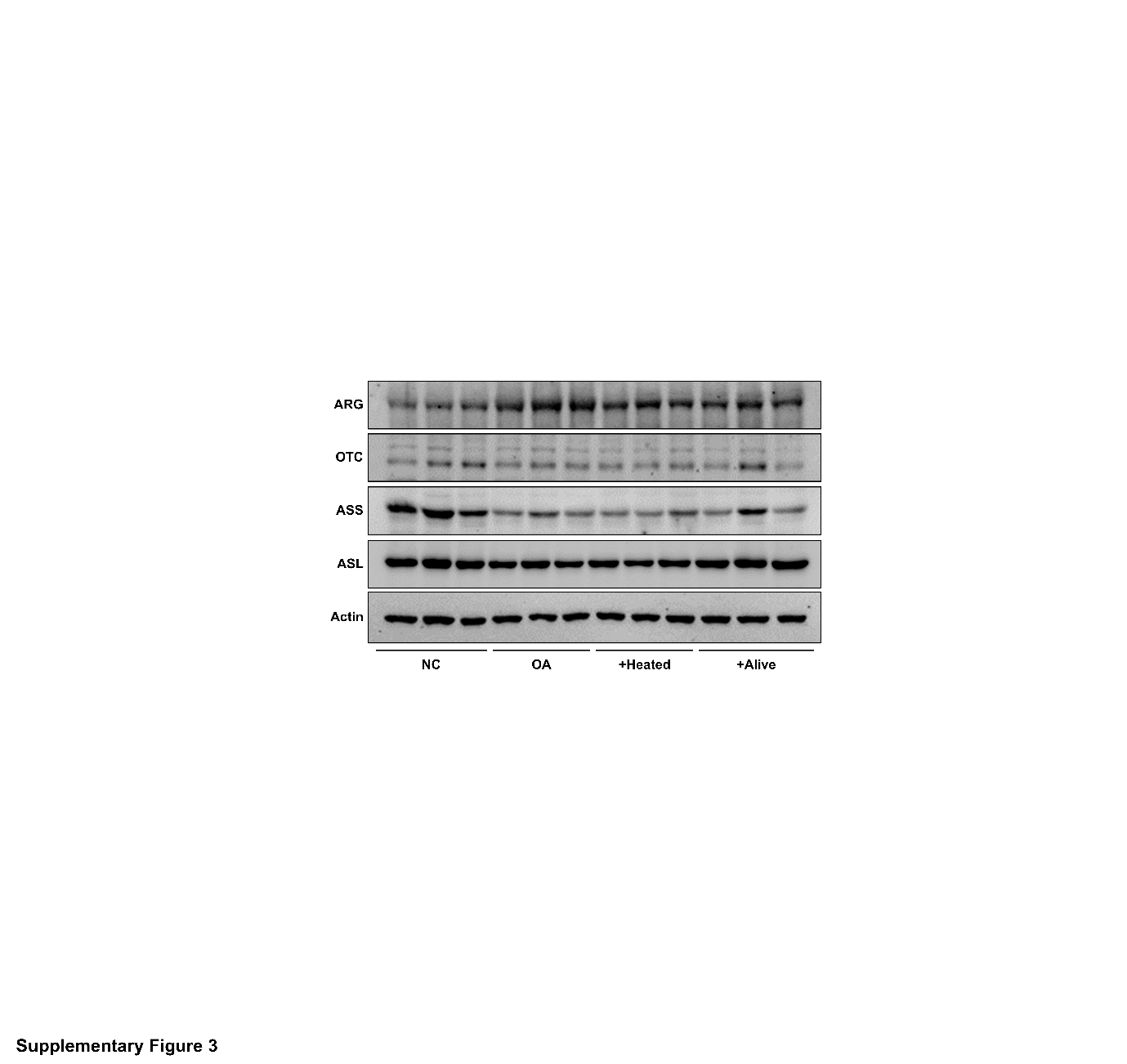
**Supplementary Figure 3:** Expression of multiple proteins involved in arginine metabolism.

ARG, arginase; ASL, argininosuccinate lyase; ASS, argininosuccinate synthase; OTC, ornithine transcarbamylase.
